
JOURNAL INFORMATION
==============================
NLM Title Abbreviation: South Med Rec
NLM Journal ID: 0027317

The Southern Medical Record

Publisher: Biomedical Journal Digitization Project

ARTICLE INFORMATION
==============================
PMCID: PMC9089476
PMID: 36023095
Article version: 1
Subjects: Editorial and Miscellaneous

The Association of Medical Editors

Print publication date: 1879 Apr 20
Volume: 9
Issue: 4
First page: 160
Last page: 160
License: This work is free of known copyright restrictions. For more information, please see PMC Back Issue Digitization.
License URL: https://creativecommons.org/publicdomain/mark/1.0/

==============================

THE Association of Medical Editors will meet in Atlanta, on Monday evening, May 5th, preceding the assembling of the American Medical Association. We anticipate much pleasure in meeting with, and forming the acquaintance of our brethren of the quill.

---

RECEIPTED.—1877—G. A. B. Hays. 1878—Drs. J. F. Pou, W. W. Coggin 6ms; M. B. Pollard; W. A. Dobbs; J. H. Payne; S. L. Singleterry; T. E. Morris; J. F. Davis; Jno. O. Brien; R. S. Hullsey; L. G. Lincecum; V. Lockheart, 6ms.; T J Parker; T. E. Collier; G. M. Rivers; R. D. Lucius. 1878-9—W. B. Maxwell; T. N. Skeen; W. R. Robison; W. T. Beall; C. M. Norwood; W. W. Farr; J. S. Green; C. W. Keller; T. J. Charlton; I N Gilmore; R. S. Powell. 1879—J. A. Holloway; C. P. Sanders; A. L. Hawkins; R. E. Hntchins; Wm. Whitehead, Lanier & McCabe; J. E. Carter, to July 1st.; J. W. Burt; P. S Anderson, W. Culpepper; J. C. McCoy; John J. Gage; J. F. Earnest, 6 ms.; M. T. Bell; D. J. Parsons; J. C. Bivings; J. T. and J. F. Alford; E. Wheeler; A. J. Kobb; J. M. Strong; Jos. Underwood; J. C. Boozer, P. Taylor; W. H. Stewart, 6 ms; Tho. H. Roberds; R. J. Tofbert; J. W. Thomas; F. J. Thorpe; T. C. Powell; T. E. Pennington, 6 ms. John Elsner; Dr. Brownlee; J. W. Harris; W. E. Brock; A. F. Fricks; J. T. Persons; Tho. B. Meacham; K. H. Davis; J. C. Wallace; S. N. Bruce; C. J. Burroughs; J. A. Fields; W. E. Saterfield; J. I. Grover; Louis Hadden; T. A. Cook; J. F. Davis; A. H. Sellers; S. Y. Carter; F. M. Bledsœ; E. H. McGehee; L. D. Webb; C. M. Gibson; Booth & Jarrett; E. R. Young; J. W. Price; S. A. Berry; W. W. Elrod; R. A. Sells; E. A. Anderson; J. L. Selman; H. Allison; M. J. Luster; R. A. Shimssock; M. B. Miller; J. K. Davidsons; F. M. Rushing; B. E. Clark; C. M. Bold s; H. C. Walkup; J. D Johnson; S. M. Cummings; J. D. Harwell; M. E. Demaret; E. H. Parham; Holderness & Robertson; D. S. Aswell; A. C. Simonton; S. H. Dilburn; C. Wall; J. G. Donald; J. W. Baker; J. A. Agnew; A. W. Agnew; W. F. Morrison; J. W. Ethridge; J. W. Bunt; T. E. Morris; S. V. Parker; T. C. Davis; M. L. Barron; C. T. Coleman; P. P. Terry; E. F. Sumerall. 1879-80—J. E. Trippe.

---

### SPECIAL NOTICES.

WE claim that a no less important discovery than that of Phosphorus by Brandt in 1669, is its preparation by William R Warner & Co., in Pillular form in its elementary state by the process we alone employ.

While phosphorus had long been regarded as a valuable remedy, the methods employed for its administration were so disgusting to the patient as to render its general use almost impossible.

The Pills prepared by us are harmless in their administration, yet most potent in their effect, the elements being thoroughly diffused and subdivided and protected from oxidation.

We present the practitioner a variety of twenty-four combinations, thus enabling him to use this valuable remedy in every variety of disease to which it is applicable.

WM. R. WARNER & CO.

**Geo. J. Howard & Bros.. Drug Establishment in Atlanta.**—Physicians visiting the city, desiring to look at or purchase medicines, should not fail to call at the Wholesale House of Geo. J. Horward & Bros., 293, Alabama street. The Senior and managing partner has been long identified with the Drug trade of this city. They have recently purchased an entirely new stock, in the most favorable state of the Drug market. They have a fine assortment of Surgical and Obstetrical Instruments, Pocket Cases, etc.

**Convenience of Empty Capsules.**—Many drugs, among which we may note *roots, gums, emetics, capsicum, etc.*, which—either from the more immediate effect to be produced, or from special action to be desired—the physician proposes to administer in the *crude* or *powdered* state, in preference to any form of preparation, are practically debarred from use in certain cases on account of their physical properties (appearance, odor, taste,) and the difficulty experienced in swallowing them.

To be able to easily disguise these features of a remedy at the bedside of a patient, at a time and place when he cannot employ the assistance of a pharmacist, is a great desideratum to the practitioner.

*Our Capsules* will fully supply this want; a few trials will demonstrate all their advantages. PARKE, DAVIS & CO., Detroit.

**Wine and Cake for Evenings.**—Alfred Speer, of New Jersey, it should be known is one of the most extensive producers and dealers in Pure Wines and Brandies in the United States. He makes a Superior Port Wine, which took the highest Premium at the Centennial, known as Speer's Port Grape Wine. It is sold by Druggists.

**Atlanta Hotels.**—The Kimball House in Atlanta, is said to be the largest and best equipped Hotel in the Southern States.

**House and Lot in Atlanta for Sale.**—A good lot, and in a locality where there are not too many Doctors. A two story house containing eleven rooms, and the usual outbuildings. Price $3.500, half cash balance on long time, with interest. A liberal discount for an all cash payment. It is a good time now to buy. Atlanta property, as is conceded by all, is at its lowest figure—and will steadily advance in the future. For particulars enquire of DR. R. C. WORD, Atlanta, Ga.
